# Cemented Modular Bipolar Hemiarthroplasty for Displaced Femoral Neck Fractures in the Elderly

**DOI:** 10.7759/cureus.74604

**Published:** 2024-11-27

**Authors:** Muhammad Moaz, Sher Afgan, Irfan Ahmad, Hammood Ur Rehman, Jawad A Chishty, Aatif Aslam, Muhammad Ibrahim

**Affiliations:** 1 Trauma and Orthopedic Surgery, Services Hospital Lahore, Lahore, PAK; 2 Trauma and Orthopedic Surgery, Jinnah Hospital, Lahore, Lahore, PAK

**Keywords:** bipolar hemi-arthroplasty, cemented hip hemiarthroplasty, displaced femoral neck fracture, elderly trauma, neck of femur fractures

## Abstract

Objective

To determine the outcomes of cemented modular bipolar hemiarthroplasty for displaced femoral neck fractures in the elderly.

Methodology

This prospective study involved 102 elderly patients with clinically and radiologically confirmed displaced femoral neck fractures and was conducted in the Department of Trauma and Orthopedic Surgery, Unit-1, Services Hospital, Lahore. Cemented bipolar hemiarthroplasty was performed on all patients. Preoperative variables included gender, laterality, and age. Postoperatively, the Harris Hip Score (HHS) at set follow-up intervals was measured to assess functional outcomes. Data analysis was conducted using JASP Team (2024), JASP (Version 0.19.0) (Computer software). Normality tests were applied to check for data distribution. As the data was non-parametric, further nonparametric tests for correlation and significance were applied.

Results

The study included 56 males and 46 females, with a mean age of 65.45 years. The majority of the procedures were left-sided (55). Cemented modular bipolar hemiarthroplasty was performed for displaced femoral neck fractures. The mean HHS at 6 weeks, 3 months, and 6 months follow-up was 78.15, 83.49, and 88.76, respectively. Complications were observed in 14 (13.7%) patients, with superficial surgical site infections (SSIs) being the most common. No cases of hip dislocation or acetabular erosion were reported during the follow-up.

Conclusion

The study concludes that cemented modular bipolar hemiarthroplasty provides better functional outcomes for elderly populations with osteoporotic bones who tend to have a relatively sedentary lifestyle.

## Introduction

Fractures of the hip are very common. According to estimates from a study, there were 36,524 hip fractures among Pakistani adults over 50 in 2015. This number is expected to rise by 214% to 114,820 by 2050 [1]. These fractures are particularly common in older individuals, who often face challenges related to sensory impairments, balance issues, and gait disturbances. All these factors contribute to an increased number of elderly people with a history of falls. In addition to these factors, there is an increased incidence of osteoporosis, which can lead to multipart and displaced fractures of the neck of the femur (FNF) with instability [2]. FNF are becoming a global challenge, especially in the elderly. Approximately 1.5 million hip fractures occur each year worldwide, with projections suggesting a potential increase to 6.26 million by 2050 [3, 4]. These fractures have been a cause of high morbidity and mortality and other health-related complications. There are different options for the treatment of displaced FNF, which include open reduction internal fixation (ORIF) with unipolar hemiarthroplasty (UHA), bipolar hemiarthroplasty (BHA), or total hip arthroplasty (THA) [4]. In the elderly, non-displaced femoral neck fractures can be treated with closed reduction and fixation with cannulated screws. Displaced femoral neck fractures in the elderly are managed with hemiarthroplasty if the baseline activity level is low, while THA is recommended for active individuals [5].

Surgeons adopt numerous ways to access the hip joint. There is considerable controversy over the treatment strategy for displaced femoral neck fractures. It is debatable whether total hip replacement or BHA is the better option, as well as whether bipolar or UHA is preferable. There is no discernible difference in the long-term results for BHA in terms of mortality, significant complications, and reoperation rates. Despite being more expensive, BHA is preferred over UHA in terms of functional success [6, 7]. The use of cemented versus non-cemented BHA is likewise controversial. Despite its functional benefits, cemented BHA is linked to concerns about heterotopic ossification, prolonged operating times, and intraoperative blood loss [8].

It has been observed that the approach used depends on the surgeon’s preference according to his experience and training [9]. This study aims to assess the functional outcomes of displaced fractures of the neck of the femur in the elderly managed with cemented BHA using the Hardinge approach. The functional outcome will be assessed according to the Harris Hip Score (HHS) developed by Harris in 1969. The HHS was developed to determine the results following hip surgery and assess various disabilities in an adult population. It has a total of one hundred points, which include pain, function, deformity, and motion evaluation [10].

Due to a lack of local literature on cemented modular BHA for displaced neck of femur fractures in the elderly, this study aims to contribute valuable insights into the effectiveness of cemented modular BHA in managing these fractures, offering a basis for informed decision-making in orthopedic practice. Through the assessment of functional outcomes using the HHS, the research seeks to enhance the understanding of post-surgical results and contribute to improved patient care.

## Materials and methods

This prospective study included 102 patients and was conducted with approval from the Institutional Review Board of the Services Institute of Medical Sciences, affiliated with Services Hospital Lahore, under approval number IRB/2023/1088/SIMS. Participants presented to the ED at Orthopedic Unit-1 of Services Hospital Lahore from March 20, 2023 to December 31, 2023 with a clinical and radiological diagnosis of displaced fracture of the femoral neck. Following informed consent, a detailed history and clinical examinations were performed.

Data collection

During data collection, inclusion and exclusion criteria were strictly adhered to for the enrollment of patients. Patients aged 60 years and above of both genders and American Society of Anesthesiologists (ASA) categories 1 and 2, with fracture types 3 and 4 according to the Garden classification, were selected and included in this study. Exclusion criteria included patients with pathological fractures other than those related to senile and post-menopausal osteoporosis, those on conservative management for other ailments, those unwilling to comply with follow-up plans, and those with any medical, neurological, or psychiatric illness that could have hampered the results.

A total of 102 patients were included in the study of cemented modular bipolar hemiarthroplasty. Age, gender, and laterality were documented on a predesigned proforma, and postoperative follow-up was planned at 6 weeks, 3 months, and 6 months. The Harris Hip Score was recorded at these predetermined follow-up intervals. Data analysis was performed using JASP Team (2024), JASP (Version 0.19.0) [Computer software]. Normality tests were applied to check for data distribution. As the data was non-parametric, Spearman correlation tests were applied to measure the correlation between age and Harris Hip Score at different time intervals. The Mann-Whitney U test was applied to compare different age groups in terms of Harris Hip Score at various intervals. A p-value of <0.05 was regarded as statistically significant.

Surgical technique

All patients underwent surgery under spinal anesthesia, following local guidelines. An intravenous antibiotic was administered 1 hour before the expected start time of surgery. All procedures were performed using the Hardinge Lateral approach. The patient was placed supine at the edge of the operating table, and a posteriorly directed lazy-J incision was made. The fascia lata was divided along with the skin and retracted anteriorly. Retraction of the gluteus maximus to the posterior side exposed the insertion of the gluteus medius and vastus lateralis. The incision was made carefully, protecting the superior gluteal nerve, to expose the anterior capsule of the hip joint, followed by a capsulotomy. Thereafter, external rotation was achieved to expose the head, followed by retrieval with a corkscrew. The proximal femur was exposed using a trochanteric reamer to remove residual portions from the lateral aspect of the neck. Access to the center of the canal was achieved using a tapered reamer to locate the medullary canal while maintaining centralization. The broach was inserted while maintaining the native femoral neck antiversion around 10-15 degrees using the axis of the flexed tibia or transepicondylar line as a crude reference point. Broaches were inserted, impacted, and extracted, progressively increasing in size while maintaining the correct axial alignment. Graduated canal reamers were used when required to adjust the largest size broach proximally, taking care to avoid over-reaming. An anticipated cement mantle thickness of 2 to 5 mm proximally and 2 mm distally was considered satisfactory. Countersinking the final broach slightly below the provisional femoral neck cut, selection of the templated neck length, and assembly of a trial component were done. The trial reduction was performed while assessing limb length and rotational stability. The depth of insertion of cement was measured at this point. After finalizing the final component and assessing limb length and stability, the removal of the trial component along with the dislocation of the head was achieved. Proximal exposure of the femur was regained, and occlusion of the femoral canal with a bone impactor distal to the anticipated length was done. Reinsertion of the broach of final trial components ensured the appropriate size. Thorough irrigation of the femoral canal was performed to remove any residual debris. The canal was dried using a tampon sponge with suction. The implant was opened, and the insertion of gentamicin-loaded polymethylmethacrylate (PMMA) cement was performed in the early dough phase at a predetermined level using an injecting gun and tapped with the broach or trial assembly to achieve final stability. Vacuum cement mixing was achieved and injected with a gun in a retrograde fashion, taking care not to allow any air, using a Nelaton placed inside the canal attached to the vacuum, which was pulled slowly as the cement filled the canal. Pressurization of the cement was done with a proximal sealer. Removal of the pressurization device was followed by filling any void created. Insertion of the Muller-type femoral component was done in the medium dough phase. Initially inserted manually and then impacted with an impactor device. Removal of any excess cement was done from the proximal femur after the late dough phase. The meticulous removal of all cement debris in the surrounding wound area was done carefully. Careful cleaning and drying of the taper was done, and assembly of the modular femoral head with a single blow using a plastic-capped impactor was achieved. The reduction was achieved, hemostasis was secured, and irrigation was done before the final closure. The final closure of layers was done in reverse order.

An immediate postoperative rehabilitation plan was given to all the patients. Patients were discharged from the hospital as weight-bearing as tolerated unless contraindicated. The goal of weight bearing as tolerated (WBAT)-assisted rehabilitation after bipolar hemiarthroplasty was early functional independence. Patients were urged to use an appropriate assistive device for safe transfers, bed mobility, and gait training during the first three days following surgery to reduce discomfort, edema, and inflammation. Post-operative precautions and a home exercise regimen intended to preserve mobility and enhance the range of motion (ROM) were the main topics of patient education. The goals of outpatient physical therapy during the motion phase (Weeks 1-6) were to strengthen the hip girdle muscles, especially the hip abductors, extensors, and core stabilizers, restore range of motion, and reduce swelling. Together with gait and functional training, proprioceptive and endurance training facilitated the shift to pain-free, unaided walking. By Weeks 6-12, the emphasis turned to strengthening the lower extremities and getting back to light functional and leisure activities like walking. The goal of advanced strengthening (Weeks 12-16) was to enhance proprioception, strength, and endurance to safely resume daily living activities and leisure activities. This procedure, customized to the patients' individual needs and available resources, ensured a safe and effective recovery. Follow-up was planned at 6 weeks, 3 months, and 6 months, respectively, where the Harris Hip Score was measured and documented. Any immediate and late postoperative complications were documented.

## Results

A total of 102 consecutive patients were included in this study: fifty-six (54.9%) were male and forty-six (45%) were female. The mean age was 65.45 ± 5.044 (CI 64.46 - 66.4). The average duration from fracture to surgery was 6.4 ± 1.9 days. All patients underwent cemented bipolar hemiarthroplasty after a femoral neck fracture. Forty-six patients (45%) sustained Garden type 3 injuries, and fifty-six (54.9%) sustained Garden type 4 injuries. All were community ambulators prior to injury. Ninety-five patients (93.1%) presented after a fall, and seven (6.8%) had fractures resulting from road traffic accidents. Fifty-five patients (53.9%) had a fractured femoral neck on the left side, while forty-seven (46%) had fractures on the right side (Table 1).

**Table 1:** Patient demographics and baseline characteristics for 102 participants.

Baseline characteristics		n (%)
Gender	Male	56 (54.9)
	Female	46 (45)
Laterality	Left	55 (53.9)
	Right	47 (46)
Reason of injury	Direct fall	95 (93.1)
	Road traffic accident	7 (6.8)
Garden classification	Type 3	46 (45)
	Type 4	56 (54.9)

Cemented modular bipolar hemiarthroplasty was performed for displaced femoral neck fractures within ten days (mean: 6.48 days) after the injury. Complications were observed in 14 (13.7%) patients, including superficial and deep surgical site infections, the most common, followed by hematoma formation and urinary tract infections. Three patients died postoperatively, with the cause of death attributed to all-cause mortality. No deaths were directly related to surgical complications (Table 2).

**Table 2:** Post-operative complications (n=102).

Complication type	n (%)
Deep surgical site infection	2 (1.96)
Superficial surgical site infection	7 (6.86)
Hematoma	2 (1.96)
Urinary tract infection	2 (1.96)
Mortality	3 (2.94)

All other patients underwent a normal course during their hospital stay. Patients with deep surgical site infections (SSIs) were managed in the operating theater with the removal of superficial and deep stitches, lavage of the wound with copious amounts of normal saline, and closure of the fascia with a Redivac drain in situ, along with local gentamicin. Superficial wounds were kept open, and IV antibiotics were commenced immediately. This was followed by daily sterile dressing changes. Both patients had successful outcomes with implant retention.

The functional outcome of the hip surgery was evaluated at different follow-up periods with the Harris Hip Score (HHS). Follow-ups were conducted at 6 weeks, 3 months, and 6 months for all patients. The descriptive statistics provide valuable insights into the progression of measurements or scores over three time intervals: 6 weeks, 3 months, and 6 months. With 99 valid observations at each time point, the data exhibits consistency in sample size. The median values increase steadily from 78 at 6 weeks to 84 at 3 months and further to 90 at six months, indicating a potential upward trend over time. Similarly, the mean values show a corresponding increase from 78.15 ± 4.99 (CI 95% 77.15-79.15) to 83.49 ± 4.5 (CI 95% 82.59-84.39) and then to 88.76 ± 3.2 (CI 95% 88.11-89.41) across the same intervals. Note, the standard deviation decreases from 6 weeks to 6 months, suggesting a reduction in the dispersion of data points as time progresses. Additionally, the range diminishes from 25 to 14, signifying a narrowing spread of values. These findings collectively imply a possible improvement and progression in the HHS over the specified time span, supported by the consistent increase in median and mean values alongside a reduction in variability (Table 3).

**Table 3:** Descriptive statistics of age and Harris Hip Score measured at different intervals. a. Confidence interval b. Harris Hip Score c. Interquartile range d. Mean absolute deviation.

Statistical value	Age	HHS ^b ^6 weeks	HHS ^b^ 3 months	HHS ^b ^6 month
Valid	102	99	99	99
Missing	0	3	3	3
Median	64.000	78.000	84.000	90.000
Mean	65.451	78.152	83.495	88.768
95% CI ^a^ Mean Upper	66.442	79.147	84.399	89.417
95% CI ^a ^Mean Lower	64.460	77.156	82.591	88.118
Std. Deviation	5.044	4.991	4.532	3.257
MAD ^d^	3.000	2.000	4.000	2.000
IQR ^c^	7.750	4.000	7.000	4.500
Variance	25.438	24.905	20.538	10.609
Skewness	0.984	-0.411	-0.612	-0.712
Std. Error f Skewness	0.239	0.243	0.243	0.243
Kurtosis	0.291	0.468	0.603	-0.178
Std. Error f Kurtosis	0.474	0.481	0.481	0.481
Shapiro-Wilk	0.891	0.961	0.948	0.925
P-value f Shapiro-Wilk	< .001	0.005	< .001	< .001
Range	20.000	25.000	22.000	14.000
Minimum	60.000	65.000	70.000	80.000
Maximum	80.000	90.000	92.000	94.000
25th percentile	61.250	76.000	81.000	86.500
50th percentile	64.000	78.000	84.000	90.000
75th percentile	69.000	80.000	88.000	91.000

A non-parametric alternative to the independent sample t-test, the Mann-Whitney U-test, was applied to check for significant differences between two age groups (60-70 and 70-80) across all time intervals during which data was collected. There is a significant difference in the HHS at 6 months between the two age groups with a p-value of 0.024 (Table 4).

**Table 4:** Significance level between age groups across all time intervals using the Mann-Whitney U-test. a. Harris Hip Score b. P-value <0.05 indicates significance​​​​​​​.

Time Interval	P-value
HHS ^a^ 6 weeks	0.426
HHS ^a ^3 months	0.539
HHS ^a ^6 months	0.024^b^

There is a negative correlation between age and HHS measured across all time points-6 weeks, 3 months, and 6 months. The increasing age is inversely related to the functional status. The decreasing functional status is attributed to the symmetrical pattern of negative correlation across all intervals (Table 5).

**Table 5:** Spearman’s correlation of age with Harris Hip Score at different time intervals. a. Harris Hip Score b. Conditioned on variable: duration of fracture (days). c. P-value <0.05 indicates significance.

Time Interval	Spearman's rho ^b^	P-value
HHS ^a ^6 weeks	-0.198	0.051
HHS ^a ^3 months	-0.205	0.043
HHS ^a^ 6 month	-0.228	0.024^c^

The overall effect of time after treatment and operation on hip functionality was also evaluated using Spearman’s correlation test. It was evident from the test that the Harris score improved over time. A statistically significant value of p = 0.002 was found when comparing the Harris scores from 6 weeks to 6 months (Table 4). This shows that there was significant improvement in the range of motion of the hip and overall hip functionality at the end of the half-year follow-up.

## Discussion

The goal of our study was to evaluate the functional outcomes in patients receiving cemented bipolar hemiarthroplasty for displaced femoral neck fractures and to determine whether the bipolar prosthesis would result in improved patient outcomes over time. Our results showed favorable hip function outcomes six months postoperatively.

Multiple studies have compared the functional outcomes of hips using unipolar or bipolar arthroplasty, each weighing their merits and demerits. Conventional unipolar hemiarthroplasty using Austin Moore or Thompson prostheses represents a cost-effective solution for addressing femoral neck fractures. Nonetheless, it is accompanied by recognized complications such as thigh pain, stem loosening, acetabular erosion, and protrusio [2, 3]. Bipolar hemiarthroplasty is designed to mitigate the issues associated with its unipolar counterpart.

Hedbeck CJ et al. conducted a study involving 120 patients who were randomly assigned to receive either unipolar or bipolar hemiarthroplasty. Notably, at the one-year follow-up, acetabular erosion was observed in 20% of patients in the unipolar group compared to 5% in the bipolar group [11]. Recent meta-analyses of randomized control trials have further enriched our understanding of these outcomes. Jia Z et al. affirmed comparable or superior results in hip function, hip pain, and quality of life for patients treated with bipolar implants [6]. Similarly, Yang B et al. reported an acetabular erosion rate of 5.5% in the unipolar group compared to a lower rate of 1.2% in the bipolar group [9]. On the other hand, Liu Y et al. did not observe a statistically significant difference between the two types of implants [4]. In a recent study by Khan AQ et al., functional outcomes in terms of the HHS were better in the bipolar group at three months follow-up, but no significant difference in functional outcome was found between the two groups at 6, 12, and 24 months (80.9 ± 2.8 vs. 82.0 ± 2.5, p-value > 0.05; 83.1 ± 2.2 vs. 83.2 ± 1.2, p-value > 0.05; 85.5 ± 2.4 vs. 85.2 ± 2.8, p-value > 0.05, respectively) [12]. The functional score of the bipolar group in this study is comparable with the score of our study at 6 weeks (78.15 ± 4.99, CI 95% 77.15-79.15), 3 months (83.49 ± 4.5, CI 95% 82.59-84.39), and 6 months (88.76 ± 3.2, CI 95% 88.11-89.41) follow-ups. A higher score in our study may indicate the need for longer follow-up and a larger number of study subjects to provide more insight.

The overall complication rate in our study was 13.7%, which is lower than the 34% complication rate seen in the bipolar group in a recent study [12]. Hedbeck reported a 22% mortality rate in his study’s bipolar group, attributed to a higher selection of males, known to have increased mortality rates [13]. The most frequent complication in our group was surgical site infection (SSI), observed in 8.8% of the patients, followed by hematoma and UTI. Superficial infections were treated by opening the wound to the fascia, lavaging with normal saline, and leaving it open for secondary healing and delayed closure, supplemented by daily aseptic dressing and IV antibiotics. For deep infections, the wound was reopened to the implant site for thorough lavage and removal of necrotic tissue, pus, and debris, with antibiotics instilled through a drain, followed by wound closure. Recovery was uneventful. No cases of acetabular erosion were reported in our study, possibly due to the lack of long-term follow-up.

The literature indicates that dislocation rates in unipolar hemiarthroplasty can reach up to 10% [14]. Conversely, bipolar hemiarthroplasty has demonstrated a lower risk of postoperative hip dislocation, presumably due to its inherent self-centering mechanism [15]. Interestingly, our study observed zero incidents of postoperative hip dislocation. While our investigation did not yield a precise explanation for this phenomenon, it is noteworthy that such occurrences were notably absent. Modular components allow various adjustments to correct limb length discrepancies and abductor and soft tissue tension, which might have contributed to the absence of hip dislocation, but this needs further validation with long-term follow-up data.

The mean age of participants in this study was 65.4 years, likely advantageous for favorable outcomes after cemented modular bipolar hemiarthroplasty. This study showed a mean HHS of 88.77 ± 3.26, comparable to a mean HHS of 87.2 ± 9.4 in patients undergoing total hip hemiarthroplasty, with an average age of participants of 81 years. The comparison group that underwent bipolar hemiarthroplasty had a mean HHS of 79.4 ± 12.3 at 12 months [11]. The preferred choice for displaced neck of femur fractures in active ambulatory adults remains debated [16, 17]. Selecting patients from appropriate age groups can result in better outcomes. In displaced neck of femur fractures, total hip arthroplasty (THA) was not found to be superior to hemiarthroplasty (HA) in terms of clinical relevance in a two-year follow-up of patients included in the fittest cohort with a mean age of 66 years and an age range from 50-70 [18]. This subgroup analysis has opened an opportunity to explore more: the effect of different age groups and activity levels on functional outcomes in patients undergoing hip arthroplasty after a displaced neck of femur fracture.

The question of long-term complications after bipolar hemiarthroplasty in this age group needs further address. One study has shown the probability of revision surgery using the Kaplan-Meier curve to be 81.8% for bipolar hemiarthroplasty at 10 years [19]. The revision rates can be reduced with the use of cemented techniques [20]. In a recent study, THA for femoral neck fractures is associated with a higher risk of dislocation compared to bipolar HA. The risk of revision or conversion was not found to be statistically different between bipolar HA and THA [21]. One study also noted an increased dislocation rate associated with THA compared to HA, while modestly favoring THA in terms of pain score, function, and Western Ontario and McMaster Universities Arthritis Index (WOMAC) score. This study did not mention the type of hemiarthroplasty used in the comparator group [22].

Hemiarthroplasty conversion to total hip arthroplasty (HA to THA) is usually done for aseptic loosening, prosthetic arthritis, periprosthetic fractures, and dislocations. HA to THA and revision THA showed comparable results in terms of operative time and estimated blood loss. The parameters like major complications are significantly less in HA to THA compared to revision THA. This favors the use of hemiarthroplasty over THA in a younger age group with significantly better outcomes if the need for THA arises later [23]. The cemented technique has its associated pros and cons. Cemented bipolar hemiarthroplasty is associated with increased operative time, increased risk of heterotropic ossification, and postoperative infection rate compared to the non-cemented technique. Regarding the obvious advantages, cemented bipolar hemiarthroplasty is associated with fewer iatrogenic fractures and decreased thigh pain [24]. This aspect must be kept under consideration when selecting an ideal technique. More studies are needed to focus on patient-reported outcome measures (PROM) besides the clinical parameters.

Strength and limitation

Our study offers the distinct advantage that all patients adhered to a uniform postoperative rehabilitation protocol. While our findings are confined to short-term follow-up, they substantiate the assertion that the bipolar prosthesis yields favorable functional outcomes based on the HHS, a metric renowned for its high validity and reliability in assessing outcomes following arthroplasty. It is essential to acknowledge the limitations of our study, including the relatively brief follow-up duration, which hinders a comprehensive assessment of acetabular erosion rates and the need for revision surgery. Additionally, the study's single-center nature represents further constraints in this investigation.

## Conclusions

Our study concluded that cemented bipolar hemiarthroplasty provides excellent clinical results and enables early rehabilitation. The technique shows low rates of postoperative complications and hip dislocations, making it a suitable choice for neck of femur fractures. A longer follow-up is necessary to explore deeper details and long-term outcomes to make informed decisions regarding the mentioned procedure.
